# Sarcopenia and the frailty progression among Chinese: a longitudinal study

**DOI:** 10.3389/fpubh.2025.1551282

**Published:** 2026-01-05

**Authors:** Xingyu Mu, Xia Wang, Qingyue Zeng, Xijie Yu, Shuangqing Li

**Affiliations:** 1 General Practice Ward, International Medical Center Ward, General Practice Medical Center, National Clinical Research Center for Geriatrics, West China Hospital, Sichuan University, Chengdu, Sichuan, China; 2 Department of Laboratory Medicine, West China Hospital, Sichuan University, Chengdu, China; 3 Laboratory of Endocrinology and Metabolism, Department of Endocrinology and Metabolism, Rare Disease Center, West China Hospital, Sichuan University, Chengdu, China

**Keywords:** sarcopenia, frailty, dynamic nature, epidemiology, CHARLS

## Abstract

**Introduction:**

Previous studies primarily focused on baseline sarcopenia status, neglecting changes over time. This study aims to explore the association between changes in sarcopenia status and frailty progression, hypothesizing that transitions to possible sarcopenia or sarcopenia increase frailty risk, while recovery to non-sarcopenia reduces this risk.

**Methods:**

Using data from the China Health and Retirement Longitudinal Study (CHARLS), sarcopenia was evaluated at baseline and after 2 years using the AWGS 2019 criteria. Frailty was assessed with a 32-item frailty index. Cox regression and linear mixed models analyzed the association between sarcopenia transitions and frailty progression.

**Results:**

Participants who developed possible sarcopenia or sarcopenia faced a 56% higher frailty risk (HR 1.56, 95% CI 1.21–2.01) and a faster frailty index increase (*β* = 0.007/year, 95% CI 0.005–0.010) compared to those remaining non-sarcopenic. In contrast, those recovering from sarcopenia to non-sarcopenia or possible sarcopenia had a 46% lower frailty risk (HR 0.54, 95% CI 0.32–0.89) and a slower frailty index rise (*β* = −0.008/year, 95% CI –0.015 to −0.002). Among individuals with possible sarcopenia at baseline, recovery to non-sarcopenia reduced frailty risk by 48% (HR 0.52, 95% CI 0.39–0.68), while progression to sarcopenia increased it by 77% (HR 1.77, 95% CI 1.07–2.94).

**Conclusion:**

Transitions in sarcopenia status significantly affect frailty risk and progression. Worsening sarcopenia heightens frailty risk, whereas recovery diminishes it. These findings highlight the value of monitoring and addressing sarcopenia in middle-aged and older adults, offering potential to enhance quality of life and lower healthcare costs through targeted interventions.

## 1 Introduction

Sarcopenia, an age-related condition characterized by the loss of muscle mass, strength, and function, is driven by factors such as inflammation and insulin resistance (1, 2). Frailty, another prevalent condition among older adults, shares similar pathogenesis involving cellular aging and endocrine metabolism regulation pathways, including endocrine resistance (3–5). The prevalence of sarcopenia varies widely, ranging from 1 to 29% in community-dwelling individuals and from 14 to 33% in long-term care settings (6). Both conditions significantly increase the risk of adverse health outcomes, including fractures, impaired mobility, decreased quality of life, and other comorbidities (7, 8). Given their overlapping mechanisms, understanding the association between sarcopenia and frailty is essential for developing effective prevention and management strategies for aging populations.

Previous studies have established that sarcopenia, including possible sarcopenia, is associated with a heightened risk of frailty (9–11). However, these studies predominantly assessed sarcopenia at baseline, neglecting its dynamic changes over time. This static approach limits insights into the trajectory of sarcopenia and its progression, as well as its potential reversibility—an emerging concept supported by evidence of recovery through targeted interventions (12, 13). Exploring changes in sarcopenia status could reveal critical biological links to frailty progression and highlight the value of integrating sarcopenia interventions into frailty management. Notably, there remains a gap in the literature regarding how such changes influence frailty risk, particularly in the Chinese population, where dynamic data on sarcopenia are scarce.

To address this gap, our study leverages prospective cohort data from the China Health and Retirement Longitudinal Study (CHARLS) to investigate the association between changes in sarcopenia status and the risk of frailty progression in the Chinese population. Our primary objective is to examine how transitions to or from sarcopenia affect frailty risk. We hypothesize that individuals transitioning from non-sarcopenia to possible sarcopenia or sarcopenia will exhibit an increased frailty risk, whereas those recovering to a less severe status will experience a reduced risk.

## 2 Methods

### 2.1 Study design and population

The CHARLS is a prospective, nationally representative cohort study conducted in China (14). Detailed information about the study design is summarized in the “Methods” section of the Supplementary data. This study used the first wave of CHARLS (2011) as the baseline and the second wave (2013) as the follow-up survey. Data from both the baseline and second survey were employed to evaluate dynamic changes in sarcopenia status, and outcomes were tracked through subsequent follow-up surveys, concluding with the fifth wave in 2020. This study is based on secondary data analysis from publicly available datasets. Clinical trial number: not applicable. CHARLS received ethical approval from the Institutional Review Board of Peking University, and informed consent was obtained from all participants.

**Figure 1:**
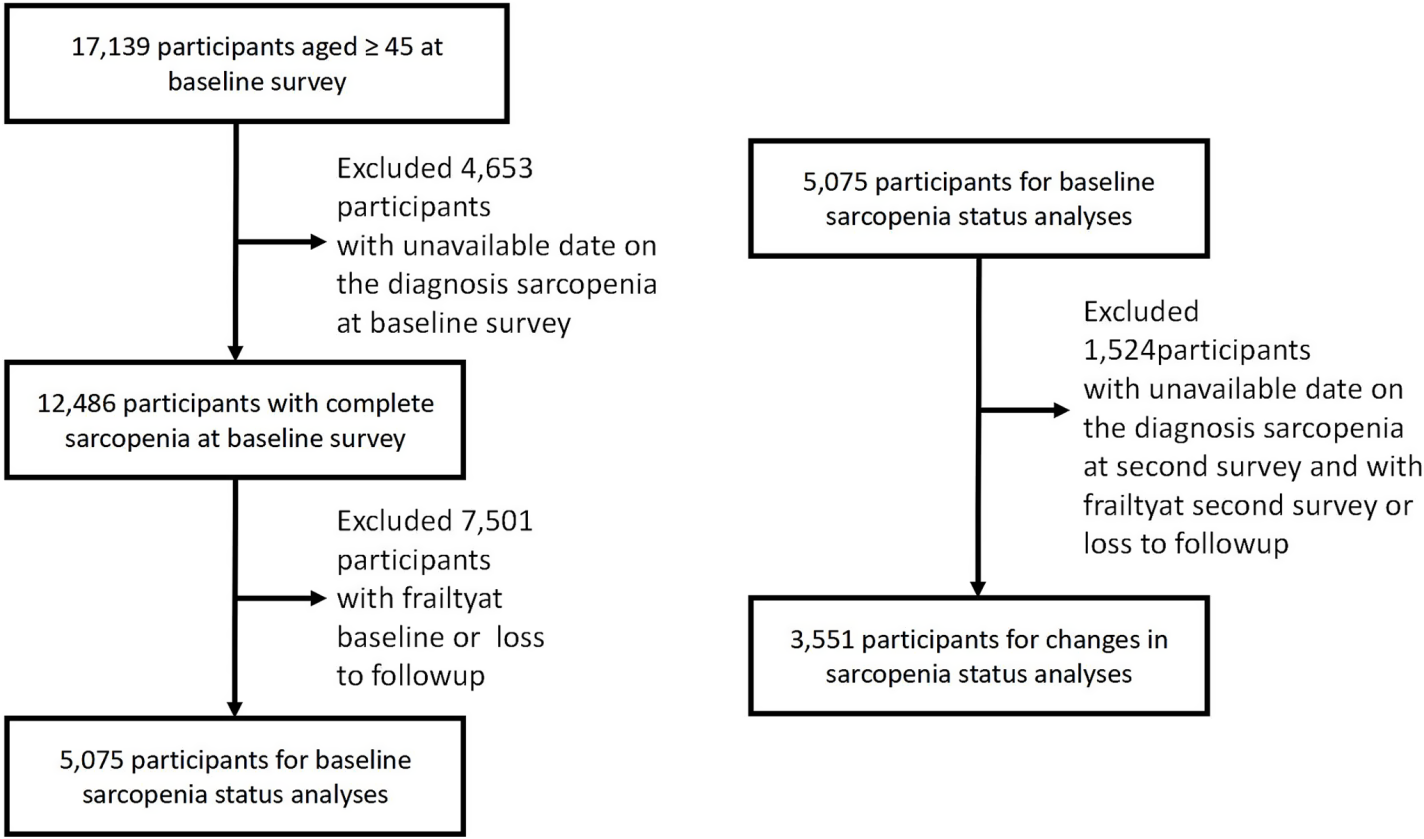
Selection process of the study population. CHARLS, China health and retirement longitudinal study.

Figure 1 illustrates the process of study population selection. Of the 17,139 participants in CHARLS, 4,653 individuals with missing baseline sarcopenia data (including appendicular skeletal muscle mass (ASM), grip strength, and physical performance) were excluded. Additionally, 7,501 individuals with missing frailty data, baseline frailty, or who were lost to follow-up were excluded. Ultimately, 5,075 eligible participants were included in the baseline sarcopenia status analysis. For the analysis of sarcopenia status changes, an additional 1,524 participants were excluded based on similar criteria, leaving 3,551 participants for the final analysis.

### 2.2 Assessment of sarcopenia status

Sarcopenia was assessed according to the 2019 algorithm of the Asian Working Group for Sarcopenia (AWGS), which evaluates three components: muscle strength, ASM, and physical performance (15). Sarcopenia is diagnosed when low muscle mass is combined with either low muscle strength or poor physical performance.

Muscle strength was measured using a YuejianTM WL-1000 dynamometer, with grip strength tested on both the dominant and non-dominant hands (15). Low grip strength was defined as <28 kg for men and <18 kg for women.

The ASM was estimated by a validated anthropometric equation in Chinese residents (16). Several studies have shown that the agreement of the ASM equation model and dual X-ray absorptiometry (DXA) was strong (16). The cut-off for defining low muscle mass was based on the sex-specific lowest 20% of the height-adjusted muscle mass (ASM/Ht2) among the study population (16, 17). In our study, body weight and height were measured using a stadiometer and a digital floor scale to the nearest 0.1 cm and 0.1 kg, respectively; the body weight and height were measured in kilograms, and centimetres, respectively (18). Finally, the ASM/Ht2 values of <7.01 kg/m^2^ in men and <5.31 kg/m^2^ in women in 2011, <7.05 kg/m^2^ in men and <5.38 kg/m^2^ in women in 2013, and <7.07 kg/m^2^ in men and <5.39 kg/m^2^ in women in 2015.

In terms of physical performance, the gait speed and the chair stand test were performed using the method described by Wu et al. (18). Further details about the definitions for sarcopenia components in the CHARLS have been described previously (15). In our study population, only 127 (2.5%) participants had severe sarcopenia in baseline. Therefore, we merged subjects with severe sarcopenia into the sarcopenia group and divided all participants into three groups: no-sarcopenia (*n* = 3,004), possible sarcopenia (*n* = 1,495), and sarcopenia (*n* = 576) in baseline.

In this study, “transition to possible sarcopenia or sarcopenia” refers to a change from non-sarcopenia to possible sarcopenia or sarcopenia, or from possible sarcopenia to sarcopenia. “Recovery” refers to a change from sarcopenia to possible sarcopenia or non-sarcopenia, or from possible sarcopenia to non-sarcopenia.

### 2.3 Ascertainment of covariates

Covariates included in the study were age, sex, marital status, educational level, residential location, smoking status, alcohol consumption, BMI (body mass index), systolic blood pressure (SBP), C-reactive protein (CRP), glycated hemoglobin (HbA1c), triglycerides (TG), high-density lipoprotein cholesterol (HDL-C), and common comorbidities such as diabetes, hypertension, and dyslipidemia. The classification criteria were as follows: marital status was categorized as “married” and “other” (including separated, divorced, never married, or widowed); educational level was grouped into “middle school or below” and “high school or above”; residential location was classified as “rural” or “urban”; smoking status was categorized as “never smokers” and “ever smokers” (including both former and current smokers); and alcohol consumption was divided into “never drinkers” and “ever drinkers.”

### 2.4 Assessment of frailty

The assessment of frailty was conducted using a 32-item frailty index (FI), encompassing many characteristics such as comorbidity, physical function, disability, depression, and cognition (19, 20). The conventional methodology for building a FI is illustrated in Supplementary Table S1. With the exception of item 32, each individual item was classified as either 0 or 1, depending on a predetermined threshold value. Several 0 indicates the absence of a deficit, whereas a value of 1 signifies the presence of a deficit. Item 32 was a continuous variable that ranged from 0 to 1, and a higher value indicated worse cognition. The 32-FI was computed for each participant by summing the existing health deficiencies and dividing the result by 32. Consequently, the FI is a continuous variable that ranges from 0 to 1. Greater values are indicative of an increased level of frailty. To optimize the sample size, we employed the median value of the associated item to estimate missing data for participants exhibiting a deficiency rate of <10% across 32 items (19). Following previous research, frailty was defined as FI ≥ 0.25 (19, 21).

### 2.5 Statistical analysis

Continuous variables were presented as mean [standard deviation (SD)] or median [interquartile range (IQR)], while categorical variables were expressed as counts (percentages). To assess the association between baseline sarcopenia status and the risk of developing frailty, Cox proportional hazards regression was employed to calculate hazard ratios (HR) and 95% confidence intervals (95% CI). Four models were used in the analysis: Model 0 was unadjusted, providing crude HR estimates; Model 1 adjusted for age and sex; Model 2 further adjusted for marital status, residential location, and educational level; and Model 3 additionally accounted for alcohol consumption, smoking, BMI, SBP, CRP, HbA1c, TG, HDL-C, and histories of diabetes, hypertension, and dyslipidemia. Covariate missing rates are summarized in Supplementary Tables S1, S2, and missing data were handled using multiple imputation by chained equations (MICE), with detailed methods provided in the Supplementary data. In addition to baseline sarcopenia, changes in sarcopenia status, was analyzed in relation to the risk of developing frailty using similar Cox regression methods. The proportional hazards assumption was tested using Schoenfeld residuals to ensure model validity (22). Covariate adjustments were based on baseline measurements, as repeated measurements for all covariates (e.g., BMI, blood pressure, or comorbidities) were not consistently available across follow-up surveys in the CHARLS dataset. This approach assumes that baseline covariates are primary drivers of frailty progression, though time-varying confounders were not explicitly modeled due to data limitations.

We also used linear mixed models to analyze the link between basic sarcopenia groups and the yearly change in the frailty index (FI) over time, based on multiple measurements, including baseline values. Such models handle repeated measurements well and use all available follow-up data to estimate the rate of change in the outcomes (23–25). We included the following terms in the models: basic sarcopenia groups, time (years since baseline), their interaction, and covariates. The interaction term showed the effect of basic sarcopenia groups on this change. We also accounted for individual differences at baseline and in the rate of change by including random effects for the intercept and time slope, using an unstructured covariance model. The analysis was also performed for changes in sarcopenia status.

Stratified analyses were conducted to explore the impact of sarcopenia status changes by sex and age groups (middle-aged: <65 years; older adults: ≥65 years) with both Cox regression. The statistical significance of interaction terms was assessed using likelihood ratio tests. Sensitivity analyses were conducted to validate the robustness of findings, including (i) reassessment of sarcopenia status during the third wave of the survey (Supplementary Figure S2), (ii) further adjustments for the use of antihypertensive and antidiabetic medications, and (iii) separate analyses for the “possible sarcopenia/sarcopenia” and “non-sarcopenia/possible sarcopenia” groups.

All statistical analyses were performed using R software (version 4.4.1). Two-sided *p*-values were calculated, and statistical significance was set at *p* < 0.05.

## 3 Results

### 3.1 Baseline characteristics of the study population

In the baseline sarcopenia status analysis, 5,075 participants (63.0% female, mean age 59.7 years) were included based on the inclusion and exclusion criteria. The baseline characteristics of these participants are detailed in Table 1. Compared to those without sarcopenia, those with sarcopenia tended to be older, had a higher proportion of women, were less likely to be married, had lower educational levels, and were more often living in rural areas. They also had lower BMI, HbA1c, UA, and HDL-c levels, but higher CRP and triglyceride levels.

**Table 1:** Baseline characteristics of participants for baseline sarcopenia status analyses.

Characteristics	Total (*n* = 5,075)	Non sarcopenia (*n* = 3,004)	Possible sarcopenia (*n* = 1,495)	Sarcopenia (*n* = 576)	*p-*value
Age, mean (SD), years	59.66 ± 8.87	57.54 ± 8.10	60.75 ± 8.56	67.84 ± 8.26	<0.0001
Sex, *n* (%)					<0.0001
Female	3,198 (63.01)	1,812 (60.32)	991 (66.29)	395 (68.58)	
Male	1,877 (36.99)	1,192 (39.68)	504 (33.71)	181 (31.42)	
Marital status, *n* (%)					<0.0001
Married	4,418 (87.05)	2,722 (90.61)	1,284 (85.89)	412 (71.53)	
Others	657 (12.95)	282 (9.39)	211 (14.11)	164 (28.47)	
Education, *n* (%)					<0.0001
Junior and below	4,730 (93.20)	2,752 (91.61)	1,412 (94.45)	566 (98.26)	
Senior and above	345 (6.80)	252 (8.39)	83 (5.55)	10 (1.74)	
Residence, *n* (%)					<0.0001
Rural	3,482 (68.61)	2,034 (67.71)	994 (66.49)	454 (78.82)	
Urban	1,593 (31.39)	970 (32.29)	501 (33.51)	122 (21.18)	
Drinking status, *n* (%)					<0.0001
Ever drinkers	1,812 (35.70)	1,171 (38.98)	464 (31.04)	177 (30.73)	
Never drinkers	3,263 (64.30)	1,833 (61.02)	1,031 (68.96)	399 (69.27)	
Smoking status, *n* (%)					<0.01
Ever smokers	1,685 (33.20)	1,050 (34.95)	453 (30.30)	182 (31.60)	
Never smokers	3,390 (66.80)	1,954 (65.05)	1,042 (69.70)	394 (68.40)	
BMI, mean (SD), kg/m^2^	23.75 ± 4.11	23.97 ± 4.07	25.10 ± 3.53	19.09 ± 1.81	<0.0001
SBP, mean (SD), mmHg	130.41 ± 21.44	129.01 ± 20.40	132.94 ± 21.96	131.13 ± 24.54	<0.0001
CRP, mean (SD), mg/L	2.50 ± 6.88	2.35 ± 6.95	2.54 ± 5.32	3.14 ± 9.53	0.04
HbA1c, mean (SD), %	5.26 ± 0.72	5.26 ± 0.70	5.30 ± 0.81	5.14 ± 0.49	<0.0001
UA, mean (SD), mg/dL	4.34 ± 1.20	4.40 ± 1.22	4.31 ± 1.16	4.12 ± 1.14	<0.0001
Triglycerides, mean (SD), mmol/L	1.32 ± 0.39	1.31 ± 0.39	1.27 ± 0.35	1.44 ± 0.40	<0.0001
HDL cholesterol, mean (SD), mmol/L	1.51 ± 1.19	1.55 ± 1.26	1.54 ± 1.19	1.25 ± 0.70	<0.0001
DM, *n* (%)					<0.01
No	4,460 (87.88)	2,626 (87.42)	1,304 (87.22)	530 (92.01)	
Yes	615 (12.12)	378 (12.58)	191 (12.78)	46 (7.99)	
Hypertension, *n* (%)					<0.0001
No	2,955 (58.23)	1,847 (61.48)	757 (50.64)	351 (60.94)	
Yes	2,120 (41.77)	1,157 (38.52)	738 (49.36)	225 (39.06)	
Dyslipidemia, *n* (%)					<0.0001
No	3,371 (66.42)	1,982 (65.98)	933 (62.41)	456 (79.17)	
Yes	1,704 (33.58)	1,022 (34.02)	562 (37.59)	120 (20.83)	

BMI, body mass index; SBP, systolic blood pressure; CRP, C-reactive protein; HbA1c, glycated hemoglobin; UA, Uric acid; HDL-C, high-density lipoprotein cholesterol; DM diabetes.

For the analysis of changes in sarcopenia status, 3,551 participants (63.1% female, mean age 59.7 years) were included according to the relevant criteria, and their baseline characteristics are presented in Table 2. Baseline characteristics of participants without imputation were also described in Supplementary Tables S3, S4, showing similar results to those in Tables 1, 2.

**Table 2:** Baseline characteristics of participants for changes in sarcopenia status analyses.

Characteristics	Total (*n* = 3,551)	Non sarcopenia (*n* = 2,133)	Possible sarcopenia (*n* = 1,038)	Sarcopenia (*n* = 380)	*P*-value
Age, mean (SD), years	59.68 ± 8.66	57.86 ± 8.10	60.42 ± 8.16	67.82 ± 8.06	<0.0001
Sex, *n* (%)					<0.01
Female	2,242 (63.14)	1,302 (61.04)	676 (65.13)	264 (69.47)	
Male	1,309 (36.86)	831 (38.96)	362 (34.87)	116 (30.53)	
Marital status, *n* (%)					<0.0001
Married	3,121 (87.89)	1,942 (91.05)	894 (86.13)	285 (75.00)	
Others	430 (12.11)	191 (8.95)	144 (13.87)	95 (25.00)	
Education, *n* (%)					<0.0001
Junior and below	3,323 (93.58)	1,962 (91.98)	988 (95.18)	373 (98.16)	
Senior and above	228 (6.42)	171 (8.02)	50 (4.82)	7 (1.84)	
Residence, *n* (%)					<0.0001
Rural	2,478 (69.78)	1,459 (68.40)	713 (68.69)	306 (80.53)	
Urban	1,073 (30.22)	674 (31.60)	325 (31.31)	74 (19.47)	
Drinking status, *n* (%)					<0.001
Ever drinkers	1,255 (35.34)	809 (37.93)	329 (31.70)	117 (30.79)	
Never drinkers	2,296 (64.66)	1,324 (62.07)	709 (68.30)	263 (69.21)	
Smoking status, *n* (%)					0.26
Ever smokers	1,179 (33.20)	730 (34.22)	332 (31.98)	117 (30.79)	
Never smokers	2,372 (66.80)	1,403 (65.78)	706 (68.02)	263 (69.21)	
BMI, mean (SD), kg/m^2^	23.77 ± 4.11	23.97 ± 4.09	25.08 ± 3.50	19.05 ± 1.76	<0.0001
SBP, mean (SD), mmHg	130.12 ± 21.30	129.01 ± 20.37	132.39 ± 21.83	130.13 ± 24.33	<0.001
CRP, mean (SD), mg/L	2.38 ± 6.11	2.28 ± 6.22	2.40 ± 4.96	2.87 ± 8.03	0.22
HbA1c, mean (SD), %	5.27 ± 0.74	5.28 ± 0.73	5.30 ± 0.83	5.13 ± 0.48	<0.001
UA, mean (SD), mg/dL	4.34 ± 1.21	4.41 ± 1.24	4.28 ± 1.16	4.14 ± 1.14	<0.0001
Triglycerides, mean (SD), mmol/L	1.50 ± 1.23	1.55 ± 1.36	1.51 ± 1.12	1.19 ± 0.58	<0.0001
HDL cholesterol, mean (SD), mmol/L	1.32 ± 0.38	1.32 ± 0.39	1.28 ± 0.35	1.45 ± 0.40	<0.0001
DM, *n* (%)					<0.01
No	3,108 (87.52)	1,851 (86.78)	904 (87.09)	353 (92.89)	
Yes	443 (12.48)	282 (13.22)	134 (12.91)	27 (7.11)	
Hypertension, *n* (%)					<0.0001
No	2,095 (59.00)	1,310 (61.42)	541 (52.12)	244 (64.21)	
Yes	1,456 (41.00)	823 (38.58)	497 (47.88)	136 (35.79)	
Dyslipidemia, *n* (%)					<0.0001
No	2,311 (65.08)	1,379 (64.65)	639 (61.56)	293 (77.11)	
Yes	1,240 (34.92)	754 (35.35)	399 (38.44)	87 (22.89)	

BMI, body mass index; SBP, systolic blood pressure; CRP, C-reactive protein; HbA1c, glycated hemoglobin; UA, Uric acid; HDL-C, high-density lipoprotein cholesterol; DM diabetes.

During the follow-up period, in the baseline sarcopenia status analysis, the median follow-up duration was 7 years, during which 929 participants developed frailty. In the sarcopenia status change analysis, the median follow-up period was 9 years, and 601 participants developed frailty.

### 3.2 Association of baseline sarcopenia status with incident frailty

The association between baseline sarcopenia status and the risk of developing frailtyis detailed in Supplementary Table S5. After adjusting for confounding factors, participants with sarcopenia had a significantly higher risk of developing frailty compared to those without sarcopenia (HR 1.61, 95% CI 1.29–2.01). Similarly, participants with possible sarcopenia also faced a significantly increased risk (HR 1.59, 95% CI 1.37–1.83). Moreover, there was a clear trend of increasing frailty risk with worsening sarcopenia severity, as indicated by the trend test (*p*-value for trend: *p* < 0.0001).

Table 3 summarizes the association between baseline sarcopenia status and FI trajectory, as analyzed using a linear mixed model. After adjusting for all variables, sarcopenia was associated with an annual FI increase of 0.008 (95% CI: 0.006 to 0.011) compared to non-sarcopenia. Similarly, possible sarcopenia was associated with a yearly FI increase of 0.006 (95% CI: 0.004 to 0.008) compared to non-sarcopenia.

**Table 3:** Association between different classes of sarcopenia status and rate of change in frailty index score.

Character	*β* (95%CI)	*P*-value
Basic sacopenia group		
Non sarcopenia	Reference	
Possible sarcopenia	0.006 (0.004, 0.008)	<0.001
Sarcopenia	0.008 (0.006, 0.011)	<0.001
Changes in sarcopenia status		
Stable non-sarcopenia	Reference	
Non-sarcopenia to possible-sarcopenia/sarcopenia	0.007 (0.005, 0.010)	<0.001
Stable possible-sarcopenia	Reference	
Possible-sarcopenia to non-sarcopenia	−0.012 (−0.017, −0.008)	<0.001
Possible-sarcopenia to sarcopenia	0.004 (−0.007, 0.016)	0.436
Stable sarcopenia	Reference	
Sarcopenia to non-sarcopenia/possible-sarcopenia	−0.008 (−0.015, −0.002)	0.015

*β* Coefficient was estimated using linear mixed models, with the positive value representing accelerated frailty. Adjusted covariates include age, gender, marriage, residence, education level, drinking, smoking, BMI, SBP, CRP, HbA1c, UA, TG, HDL-C, as well as history of DM, hypertension and Dyslipidemia. BMI, body mass index; SBP, systolic blood pressure; CRP, C-reactive protein; HbA1c, glycated hemoglobin; UA, Uric acid; TG, Triglycerides; HDL-C, high-density lipoprotein cholesterol; DM diabetes.

### 3.3 Association of changes in sarcopenia status with incident frailty

Over a two-year follow-up period, 566 participants (26.5%) who were initially free of sarcopenia progressed to a state of possible sarcopenia or sarcopenia, while 161 participants (42.3%) with baseline sarcopenia recovered to a state of no sarcopenia or possible sarcopenia (Table 4).

**Table 4:** Number and percentage of the changes in sarcopenia status.

Baseline	The second survey	Percent *n* (%)
Non-sarcopenia	Non-sarcopenia	1,567 (73.5)
	Possible-sarcopenia	426 (20)
	Sarcopenia	140 (6.5)
Possible-sarcopenia	Non-sarcopenia	552 (53.2)
	Possible-sarcopenia	449 (43.3)
	Sarcopenia	37 (3.6)
Sarcopenia	Non-sarcopenia	135 (35.5)
	Possible-sarcopenia	26 (6.8)
	Sarcopenia	219 (57.6)

The time interval between baseline and the second survey was 2 years in the CHARLS.

The association between changes in sarcopenia status and the risk of developing frailty is shown in Table 5. Compared to stable non-sarcopenia participants, those who progressed from non-sarcopenia to possible sarcopenia or sarcopenia had a significantly higher risk of developing frailty (HR 1.56, 95% CI 1.21–2.01). On the other hand, sarcopenia participants who improved to non-sarcopenia or possible sarcopenia showed a significantly lower risk of new-onset frailty (HR 0.54, 95% CI 0.32–0.89). For participants with baseline possible sarcopenia, those who recovered to non-sarcopenia had a significantly lower risk of frailty compared to those with stable possible sarcopenia (HR 0.52, 95% CI 0.39–0.68), while those who progressed to sarcopenia had a significantly increased risk (HR 1.77, 95% CI 1.07–2.94).

**Table 5:** Association of changes in sarcopenia status with risks of incident frailty.

Character	Events/*n*	Crude model	Model 1	Model 2	Model 3
		HR (95% CI)	HR (95% CI)	HR (95% CI)	HR (95% CI)
Stable non-sarcopenia	166/1567	Reference	Reference	Reference	Reference
Non-sarcopenia to possible-sarcopenia/sarcopenia	102/566	1.77 (1.39, 2.27)	1.56 (1.21, 2.01)	1.51 (1.17, 1.95)	1.56 (1.21, 2.01)
Stable possible-sarcopenia	146/449	Reference	Reference	Reference	Reference
Possible-sarcopenia to non-sarcopenia	86/552	0.43 (0.33, 0.56)	0.47 (0.36, 0.62)	0.47 (0.36, 0.62)	0.52 (0.39, 0.68)
Possible-sarcopenia to sarcopenia	18/37	1.67 (1.02, 2.72)	1.49 (0.90, 2.45)	1.45 (0.88, 2.39)	1.77 (1.07, 2.94)
Stable sarcopenia	60/219	Reference	Reference	Reference	Reference
Sarcopenia to non-sarcopenia/possible-sarcopenia	23/161	0.48 (0.30, 0.78)	0.54 (0.33, 0.90)	0.55 (0.33, 0.91)	0.54 (0.32, 0.89)

Model 1 included adjustments for age and gender; Model 2 further adjusted for marriage, residence, and education level; and Model 3 additionally adjusted for drinking, smoking, BMI, SBP, CRP, HbA1c, UA, TG, HDL-C, as well as history of DM, hypertension and Dyslipidemia. BMI, body mass index; SBP, systolic blood pressure; CRP, C-reactive protein; HbA1c, glycated hemoglobin; UA, Uric acid; TG, Triglycerides; HDL-C, high-density lipoprotein cholesterol; DM diabetes.

Further insights from the linear mixed model analysis highlight the relationship between changes in sarcopenia status and FI trajectory within this model. As shown in Table 3, after adjusting for all covariates, participants who progressed from non-sarcopenia to possible sarcopenia or sarcopenia exhibited an accelerated increase in FI (*β* = 0.007 per year; 95% CI: 0.005 to 0.010 per year) compared to those with stable non-sarcopenia. Conversely, participants with sarcopenia who improved to non-sarcopenia or possible sarcopenia demonstrated a reduced rate of FI increase (*β* = −0.008 per year; 95% CI: −0.015 to −0.002 per year). Among those with baseline possible sarcopenia, individuals who recovered to non-sarcopenia showed a significant reduction in the rate of FI increase (*β* = −0.012 per year; 95% CI: −0.017 to −0.008 per year), while progression to sarcopenia did not reach statistical significance (*β* = 0.004 per year; 95% CI: −0.007–0.016 per year).

While the annual FI increases (e.g., *β* = 0.007/year for non-sarcopenia to possible sarcopenia/sarcopenia) appear small, their cumulative effect over several years may contribute to clinically meaningful frailty progression.

### 3.4 Subgroup analyses and sensitivity analyses

In the subgroup analysis, compared to participants with stable non-sarcopenia, those who progressed from non-sarcopenia to possible sarcopenia or sarcopenia status exhibited significantly higher risks of incident frailty across all subgroups (female HR 1.79, 95% CI 1.32–2.44; male HR 1.72, 95% CI 1.13–2.60; <65 years HR 1.51, 95% CI 1.11–2.07; ≥65 years HR 1.89, 95% CI 1.25–2.88). Participants who recovered from sarcopenia to either non-sarcopenia or possible sarcopenia showed a reduction in frailty risk in men and individuals under 65 years of age (male HR 0.24, 95% CI 0.09–0.63; <65 years HR 0.08, 95% CI 0.02–0.33). However, this reduction in risk was not statistically significant in women and individuals over 65 years of age (female HR 0.65, 95% CI 0.37–1.15; ≥65 years HR 0.92, 95% CI 0.54–1.54). For participants with baseline possible sarcopenia, those who recovered to non-sarcopenia status showed significantly reduced risks of incident frailty compared to those with stable possible sarcopenia across all subgroups (female HR 0.39, 95% CI 0.28–0.54; male HR 0.53, 95% CI 0.33–0.89; <65 years HR 0.43, 95% CI 0.31–0.59; ≥65 years HR 0.51, 95% CI 0.33–0.81). Subgroup analyses are provided in Supplementary Tables S6–S8.

When re-assessing changes in sarcopenia status using data from the third survey, consistent results were observed (Supplementary Tables S9, S10). The progression to sarcopenia status continued to be associated with an increased risk of incident frailty, while recovery from sarcopenia status was linked to a decreased risk of incident frailty. Additionally, these findings remained consistent with the main analyses even after further adjustment for the use of antihypertensive and antidiabetic medications (Supplementary Tables S11, S12). When separating the possible sarcopenia/sarcopenia group and the non-sarcopenia/possible sarcopenia group (Supplementary Table S13), the results remained consistent: participants without sarcopenia who progressed to possible sarcopenia or sarcopenia had an increased risk of incident frailty, while those who recovered from sarcopenia to non-sarcopenia showed a reduced risk of developing frailty.

## 4 Discussion

### 4.1 Summary of major findings

Our findings directly address the knowledge gaps from the Introduction concerning the lack of longitudinal evidence on how sarcopenia state transitions (including recovery) influence frailty risk in Chinese older adults. In this study with the prospective cohorts, we examined the associations of baseline and changes in sarcopenia status with frailty progression. Specifically, participants with possible sarcopenia or sarcopenia exhibited higher frailty risks than non-sarcopenic individuals. More importantly, those who progressed from non-sarcopenia to possible/sarcopenia states showed elevated frailty risks, while recovery from possible/sarcopenia to non-sarcopenia significantly attenuated frailty risk—validating our hypothesis that sarcopenia reversibility modifies frailty trajectories. This provides the first population-based evidence from China that sarcopenia recovery is a modifiable factor for frailty prevention, aligning with intervention studies on sarcopenia reversibility.

### 4.2 Comparative analysis with literature

With the rapid increase in the global aging population, sarcopenia has become an increasingly common condition. Previous studies have demonstrated a strong association between sarcopenia status and frailty (9–11). In a study involving a total of 774 older adult CKD I-IV patients (>60 years of age) recruited from 29 clinical centers in China, sarcopenia was found to be independently associated with an increased risk of frailty in this population. Patients with sarcopenia, advanced age, and other risk factors should be evaluated for frailty (9). In a study of 1,538 participants from the Toledo Study of Healthy Aging, sarcopenic individuals had a higher likelihood of transitioning from robustness or prefrailty to frailty, while non-sarcopenic individuals were more likely to improve from prefrailty to robustness and remain robust. Sarcopenia was strongly associated with an increased risk of frailty progression, with odds ratios ranging from 2.34 to 4.73 (10). In our prospective cohort study further supporting the notion that sarcopenia should be considered an independent risk factor for frailty.

In addition to examining baseline sarcopenia status, our study is the first to explore the association between changes in sarcopenia status and the risk of developing frailty, an aspect not addressed in previous research. In a study comprised 4,395 individuals, with a total of 10,778 sarcopenia status assessments. Of the participants, 60.3% remained in a state of possible sarcopenia, 24.5% recovered from possible sarcopenia to no sarcopenia, 6.7% progressed to sarcopenia, and 8.5% died during the next follow-up (26). Our study confirmed previous findings on the dynamic nature of sarcopenia status in the CHARLS cohort. More importantly, we found that Transitions in sarcopenia status significantly affect frailty risk and progression. These findings warrant further investigation in larger cohort studies to better understand the relationship between changes in sarcopenia status and frailty risk. The clinical significance of the observed FI changes warrants further discussion. Annual FI increases, such as *β* = 0.007/year for participants transitioning to possible sarcopenia or sarcopenia, may seem modest. However, over a decade, this translates to an FI increase of 0.07, approaching the threshold for frailty (FI ≥ 0.25) in some individuals, particularly those with higher baseline FI values. Thus, the cumulative impact of these small annual changes can substantially elevate frailty risk over time, underscoring the importance of early interventions to prevent sarcopenia progression or promote recovery.

### 4.3 Mechanistic discussion

Sarcopenia and frailty result from interconnected disruptions in cellular energy metabolism and systemic endocrine regulation (3–5). A central contributor is the age-related dysfunction of AMP-activated protein kinase (AMPK), which impairs mitochondrial biogenesis by reducing peroxisome proliferator-activated receptor gamma coactivator 1-alpha (PGC-1α) activation, disrupts autophagy through decreased Unc-51-like autophagy activating kinase 1 (ULK1) phosphorylation, and promotes protein breakdown via overactivation of the mechanistic target of rapamycin (mTOR) pathway (27–29). This AMPK dysfunction cascade triggers systemic oxidative stress, accelerating muscle atrophy and frailty progression.

Endocrine dysregulation further exacerbates muscle vulnerability through interrelated pathways. Insulin resistance drives protein breakdown to supply energy substrates while elevating free fatty acids that suppress AMPK, creating a harmful feedback loop (30). Concurrently, reduced growth hormone/insulin-like growth factor-1 (IGF-1) signaling impairs muscle cell regeneration and increases intramuscular fat accumulation (31). Excess glucocorticoids activate muscle-specific ubiquitin ligases (MuRF1/Atrogin-1) and inhibit satellite cell differentiation (31). Notably, these pathways interact: insulin resistance worsens AMPK dysfunction, and glucocorticoids disrupt IGF-1 receptor signaling (31), amplifying the pathological interplay underlying sarcopenia and frailty.

### 4.4 Clinical and public health implications

Our study has important clinical and public health implications. First, integrating sarcopenia assessment into routine frailty management is crucial, particularly among older populations. Individuals with sarcopenia or possible sarcopenia should be prioritized as target groups for the prevention of adverse depressive outcomes. Additionally, even those without sarcopenia should be evaluated for sarcopenia-related risk factors to allow early identification of at-risk individuals. Preventive measures aimed at delaying the progression of sarcopenia could reduce the incidence of frailty in affected populations. Furthermore, given that sarcopenia is a reversible condition, the observed reduction in frailty risk among individuals who recover from sarcopenia underscores the importance of implementing effective interventions to reverse sarcopenia status. Possible sarcopenia presents a key window of opportunity for intervention, as it offers a higher likelihood of transitioning to a no sarcopenia state compared to those already diagnosed with sarcopenia (Table 3). This makes it an ideal stage for preventing frailty. Exercise plays a significant role in both sarcopenia and frailty management. Previous research has shown that exercise exerts positive effects by regulating inflammation, oxidative stress, psychosocial factors, and the neuroendocrine system (12, 32). Finally, future research is needed to generate real-world data and conduct clinical trials to explore the most effective interventions for reversing sarcopenia and assess their efficacy and safety within the context of frailty management practices. Such measures improve individual health outcomes and alleviate the societal burden of frailty-related healthcare costs and caregiver demands, fostering more sustainable healthcare systems (33).

### 4.5 Strengths

This study has several strengths. To our knowledge, this is the first study to investigate the association between changes in sarcopenia status and the risk of developing frailty. Moreover, the study utilized a large-scale, nationally representative sample, which enhances the generalizability of the findings to the general middle-aged and older population in China. Thirdly, during the follow-up period, not only were data on frailty risk collected but the FI was measured multiple times, employing various methods to analysis associations of baseline and changes in sarcopenia status with frailty progression in China. A variety of sensitivity analyses were conducted, further ensuring the robustness of the results.

### 4.6 Limitations and future research directions

There are some limitations in this study. First, consistent with prior research (34–36), certain components of frailty assessment, such as chronic disease history, were based on self-reported data, which may introduce recall bias and affect the evaluation of the relationship between changes in sarcopenia status and frailty. However, previous studies have shown that the a 32-item frailty index is a reliable screening tool for frailty, with strong internal consistency and it has been validated in the Chinese older adult population (19, 21). While more frequent surveys could theoretically provide a more precise evaluation of sarcopenia status changes, our sensitivity analyses, which included reassessment using the third survey, yielded consistent results, supporting the reliability of our findings. Thirdly, Although we adjusted for multiple confounders, there may still be residual confounding factors, such as genetic predisposition and diet, that were not accounted for. Future research should focus on addressing these important issues. Additionally, our analysis adjusted for baseline covariates but did not account for time-varying confounders, such as changes in BMI, blood pressure, or comorbidities over time, due to inconsistent availability of repeated measurements in the CHARLS dataset. This may introduce residual confounding, particularly for covariates that change dynamically with aging or interventions, potentially affecting the estimated associations between sarcopenia status changes and frailty progression. Additionally, the observational design and the dropout rate during follow-up inevitably introducing selection bias should also be taken into account when interpreting and extrapolating our results. Furthermore, our study includes only Chinese participants and uses anthropometric equations validated for this population, limiting direct generalizability to other ethnic or national groups. Differences in sarcopenia prevalence, frailty assessment methods, genetic predispositions, and lifestyle factors (e.g., diet, physical activity) may influence the association between sarcopenia status changes and frailty progression in other populations. For instance, populations with higher baseline muscle mass or different aging patterns may exhibit varying risks of frailty progression. Despite these limitations, this study contributes to advancing our understanding of the importance of changes in sarcopenia status for managing frailty.

## 5 Conclusion

This study provides novel insights into the dynamic relationship between sarcopenia and frailty by demonstrating that changes in sarcopenia status significantly influence frailty progression. Specifically, progression to possible sarcopenia or sarcopenia increases frailty risk, while recovery to non-sarcopenia or possible sarcopenia reduces this risk. These findings address a critical gap in the literature, as prior studies largely overlooked how sarcopenia evolves over time and its impact on frailty, particularly in the Chinese population.

Our findings contribute to Sustainable Development Goal by emphasizing the importance of early sarcopenia intervention and management to prevent frailty and its associated adverse health outcomes. Such measures improve individual health outcomes and alleviate the societal burden of frailty-related healthcare costs and caregiver demands, fostering more sustainable healthcare systems.

Looking ahead, future research should prioritize developing and evaluating precise prevention strategies and tailored interventions to reverse sarcopenia and mitigate frailty risk, especially in high-prevalence populations like the older adult in China. Additionally, studies assessing the cost-effectiveness of these interventions will be vital for integrating them into public health policies and clinical practices, ensuring long-term benefits for both individuals and society.

## Data Availability

The original contributions presented in the study are included in the article/Supplementary material, further inquiries can be directed to the corresponding authors.
