# Supplementary material for: Sarcopenia and the frailty progression among Chinese: a longitudinal study
Source: Front Public Health. 2026 Jan 5;13:1551282. doi: 10.3389/fpubh.2025.1551282 (PMC12812598; doi:10.3389/fpubh.2025.1551282)

**Supplemental Methods**

**Study designs of** **the CHARLS**

The China Health and Retirement Longitudinal Study (CHARLS) was a prospective cohort study conducted in China. In wave 1, a nationally representative sample of 17,708 participants was recruited from 28 provinces in 2011 via multistage probability sampling. The primary aim of this study was to recruit participants aged ≥ 45 years, but some participants aged 40 to 44 years also attended the baseline survey. All 17,708 participants underwent face-to-face interviews by the trained staff using the standardized questionnaire to collect data on sociodemographic information, lifestyles, and health-related information. Among 17,708 participants, 13,978 participants conducted anthropometric measurements to collect data on height, weight, waist circumference, hip circumference, blood pressure, grip strength, and so on. In addition, 11,847 participants provided blood samples for the laboratory test. Biochemical indicators, including total cholesterol, high-density lipoprotein cholesterol, low-density lipoprotein cholesterol, glycated hemoglobin, fasting blood glucose, and C-reactive protein were measured by the blood test. The follow-up surveys were conducted in 2013 (wave 2), 2015 (wave 3), and 2018 (wave 4) with questionnaire interviews and anthropometric measurements. Blood samples were collected in 2015 again, and biochemical indicators were measured by the blood test.

**Ascertainment of frailty**

The assessment of frailty was conducted using a 32-item frailty index, encompassing many characteristics such as comorbidity, physical function, disability, depression, and cognition(1, 2).The conventional methodology for building a frailty index is illustrated in supplemental methods table 1. With the exception of item 32, each individual item was classified as either 0 or 1, depending on a predetermined threshold value. Several 0 indicates the absence of a deficit, whereas a value of 1 signifies the presence of a deficit. Item 32 was a continuous variable that ranged from 0 to 1, and a higher value indicated worse cognition. The 32-frailty Index (32-FI) was computed for each participant by summing the existing health deficiencies and dividing the result by 32. Consequently, the FI is a continuous variable that ranges from 0 to 1. Greater values are indicative of an increased level of frailty. To optimize the sample size, we employed the median value of the associated item to estimate missing data for participants exhibiting a deficiency rate of <10 % across 32 items(1). Following previous research, frailty was defined as FI ≥ 0.25(1).

**Multiple imputation**

The missing data of covariates were imputed using the multiple imputation with chained equation(3). In the CHARLS, we only imputed the covariates in which the missing rates were less than 80% recommended by previous studies(4, 5).All eligible covariates were imputed using one imputation model which included the age, sex, education, marital status, smoking status, drinking status, body mass index, systolic blood pressure, glycated hemoglobin, high-density lipoprotein cholesterol, C-reactive protein, antihypertensive drug, and antidiabetic drug. In each cohort, we performed 5 imputations and generated 5 imputed datasets. Effect estimates were computed separately for each of the 5 datasets, and then combined according to Rubin’s rules(3).The multiple imputation was conducted using the R package "mice".

**Reference**

1. Duan L, Xiao M, Liu S, Wu Z, Chen R, Zeng R, et al. Associations between modifiable risk factors and frailty progression among individuals with pre-frailty. Exp Gerontol. 2024;194:112494.

2. Searle SD, Mitnitski A, Gahbauer EA, Gill TM, Rockwood K. A standard procedure for creating a frailty index. BMC Geriatr. 2008;8:24.

3. White IR, Royston P, Wood AM. Multiple imputation using chained equations: Issues and guidance for practice. Stat Med. 2011;30(4):377-99.

4. van Es N, Takada T, Kraaijpoel N, Klok FA, Stals MAM, Büller HR, et al. Diagnostic management of acute pulmonary embolism: a prediction model based on a patient data meta-analysis. Eur Heart J. 2023;44(32):3073-81.

5. Madley-Dowd P, Hughes R, Tilling K, Heron J. The proportion of missing data should not be used to guide decisions on multiple imputation. J Clin Epidemiol. 2019;110:63-73.

Supplementary Methods Table 1. Detailed description of the 32 items used to construct the frailty index and their corresponding cut-off values.

| No | Description of the items | Cut-off value |
| --- | --- | --- |
|  | CHARLS |  |
| 1 | Self-reported physician diagnosed hypertension | Yes = 1, No = 0 |
| 2 | Self-reported physician diagnosed diabetes | Yes = 1, No = 0 |
| 3 | Self-reported physician diagnosed heart disease | Yes = 1, No = 0 |
| 4 | Self-reported physician diagnosed stroke | Yes = 1, No = 0 |
| 5 | Self-reported physician diagnosed cancer | Yes = 1, No = 0 |
| 6 | Self-reported physician diagnosed arthritis | Yes = 1, No = 0 |
| 7 | Self-reported physician diagnosed chronic lung disease | Yes = 1, No = 0 |
| 8 | Self-reported physician diagnosed asthma | Yes = 1, No = 0 |
| 9 | Self-reported physician diagnosed any emotional, nervous, or psychiatric problems | Yes = 1, No = 0 |
| 10 | Self-reported physician diagnosed memory-related disease | Yes = 1, No = 0 |
| 11 | Self-reported vision problems | Yes = 1, No = 0 |
| 12 | Self-reported hearing problems | Yes = 1, No = 0 |
| 13 | Self-reported general health status | Poor or fair = 1, excellent, very good, or good = 0 |
| 14 | Difficulty with dressing | Yes = 1, No = 0 |
| 15 | Difficulty with bathing or showering | Yes = 1, No = 0 |
| 16 | Difficulty with eating | Yes = 1, No = 0 |
| 17 | Difficulty with getting in and out of bed | Yes = 1, No = 0 |
| 18 | Difficulty with using the toilet | Yes = 1, No = 0 |
| 19 | Difficulty with managing money | Yes = 1, No = 0 |
| 20 | Difficulty with taking medications | Yes = 1, No = 0 |
| 21 | Difficulty with shopping for groceries | Yes = 1, No = 0 |
| 22 | Difficulty with preparing meals | Yes = 1, No = 0 |
| 23 | Difficulty with doing housework | Yes = 1, No = 0 |
| 24 | Mobility: difficulty with walking 100 yards | Yes = 1, No = 0 |
| 25 | Mobility: difficulty with getting up from a chair after sitting for long periods | Yes = 1, No = 0 |
| 26 | Mobility: difficulty with climbing several flights of stairs without resting | Yes = 1, No = 0 |
| 27 | Mobility: difficulty with lifting or carrying weights over 10 pounds/jins | Yes = 1, No = 0 |
| 28 | Mobility: difficulty with picking up a coin from the table | Yes = 1, No = 0 |
| 29 | Mobility: difficulty with stooping, kneeling, or crouching | Yes = 1, No = 0 |
| 30 | Mobility: difficulty with reaching arms above shoulder level | Yes = 1, No = 0 |
| 31 | Depression: CESD-10 questionnaire | CESD-10 >=10 =1, <10 =0 |
| 32 | Cognition: (memory test score + orientation test score) **/** 14 | Continuous, ranging from 0 to 1 |

Heart disease indicates the angina, coronary heart disease, congestive heart failure, or other heart problems.

Memory-related disease indicates Alzheimer’s disease or dementia, organic brain senility, or other serious memory impairment.

Depression is evaluated using Center for Epidemiologic Studies Depression Scale (CESD). In the CHARLS, CESD-10 is used, and the total score ranges from 0 to 30. The higher score indicates more severe depressive symptoms.

The memory score is the average of words that are not recalled in the immediate and delayed word recall tasks. The memory score ranges from 0 to 10. The orientation test comprises four questions about the day of the week, the month, the date of the month, and the year. One point is given for each wrong answer, and the range is from 0 to 4.

**Supplemental Figure Legends**

**Supplemental Figure S1 Selection process of the study population**

CHARLS, China Health and Retirement Longitudinal Study

**
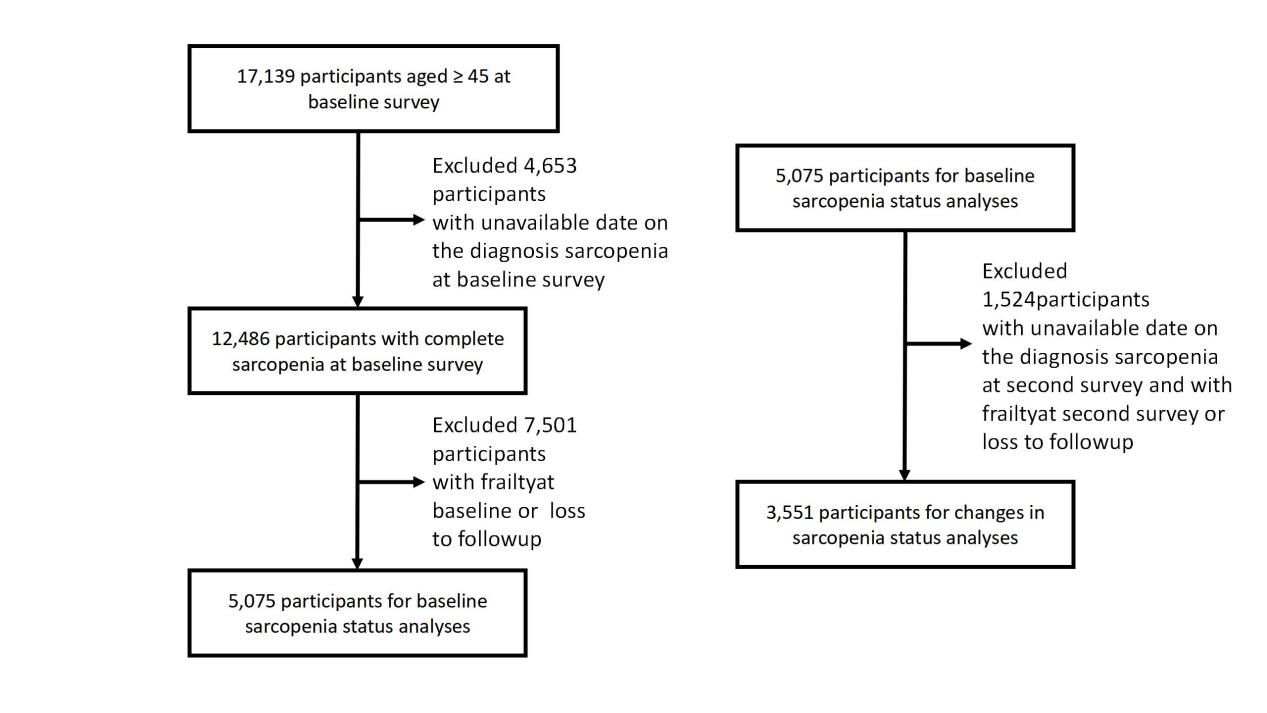
**

**Supplemental Figure S2a.** Change trajectory of sarcopeniastatus

In main analyses, changes in sarcopenia status were assessed based on the sarcopenia status at baseline and the second survey which was conducted two years after the baseline.

Because the sample sizes of non-sarcopenia to sarcopenia and sarcopenia to non-sarcopenia trajectories were small, we integrated these two trajectories into non-sarcopenia to possible-sarcopenia /sarcopenia and sarcopenia to non-sarcopenia/possible-sarcopenia.

**
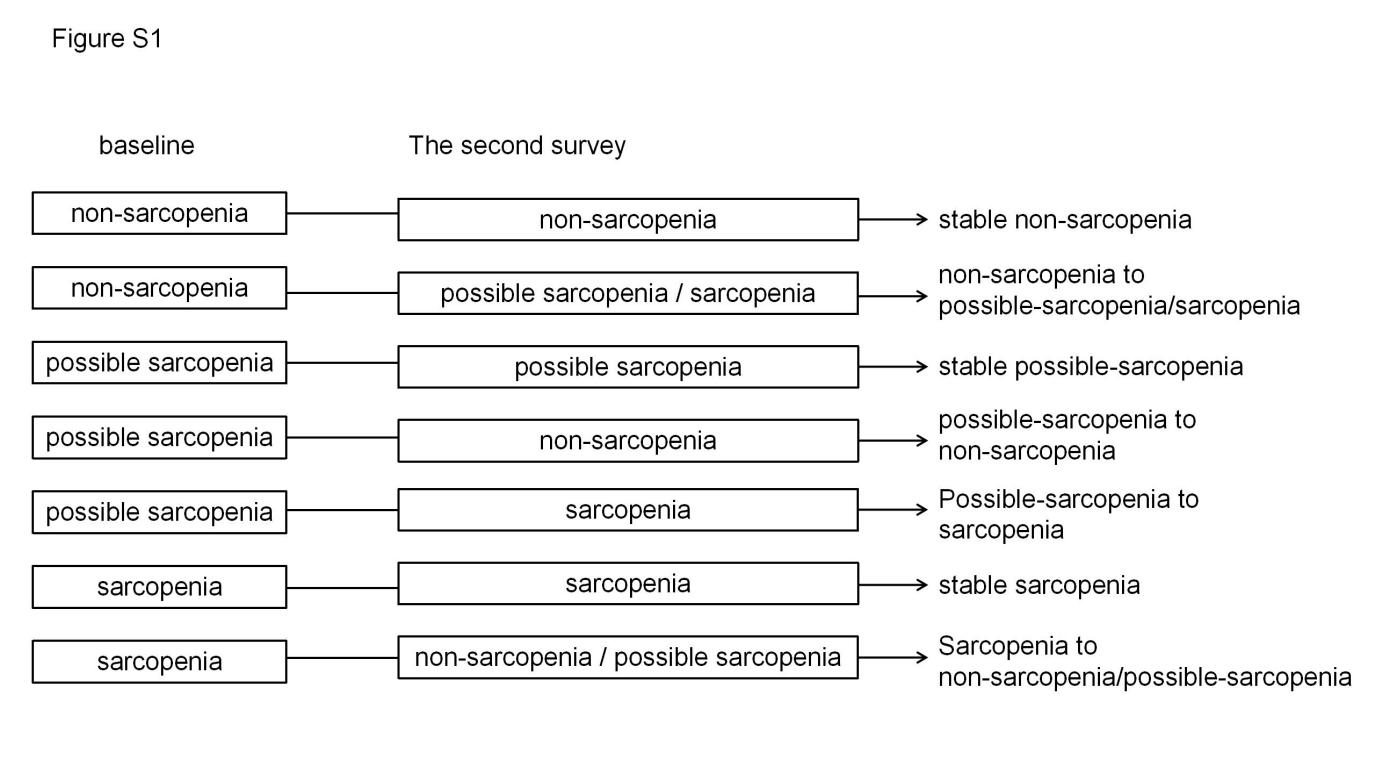
**

**Supplemental Figure S2b.** Stable change trajectory of sarcopenia status when using the third survey

In sensitivity analyses, to reduce the bias from potentially unstable changes in sarcopenia status, we used the third survey (wave 3 of CHARLS) to ensure the stability of sarcopenia changes.

Unstable changes in sarcopenia status meant that sarcopenia status changed again at the third survey, such as participants who changed from possible-sarcopenia to non-sarcopenia from baseline to the second survey, but became possible-sarcopenia again at the third survey. Participants with unstable changes in sarcopenia status were excluded from this analysis.

Stable changes in sarcopenia status meant that sarcopenia status remained stable at the third survey, such as participants who changed from possible-sarcopenia to non-sarcopenia from baseline to the second survey, and remained non-sarcopenia at the third survey. Participants with stable changes in sarcopenia status were included in this analysis. The sample sizes for this analysis were 2944 in the study


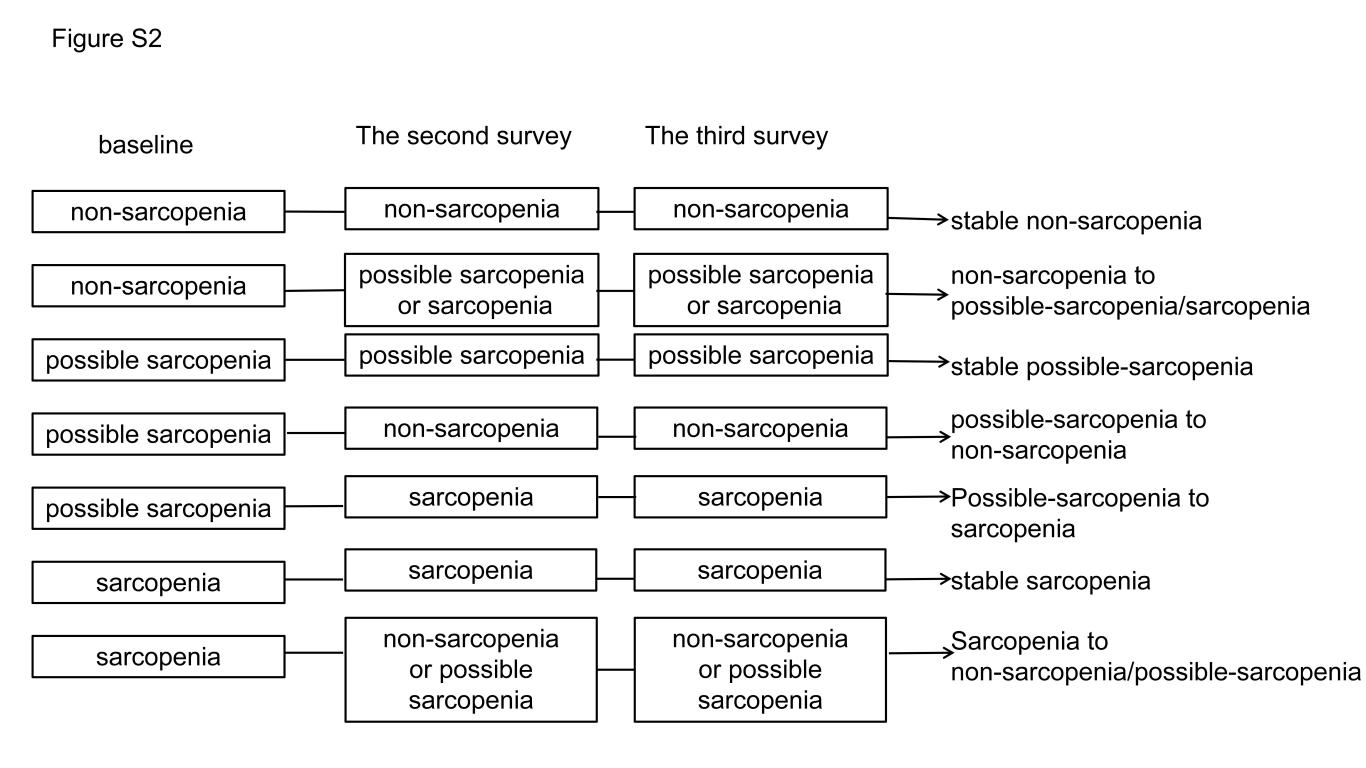

Supplement: Supplementary file 1 [file Table_1.docx]
